# Phylogenomics and plastome evolution of *Indigofera* (Fabaceae)

**DOI:** 10.3389/fpls.2023.1186598

**Published:** 2023-06-06

**Authors:** Sheng-Mao Zhou, Fang Wang, Si-Yuan Yan, Zhang-Ming Zhu, Xin-Fen Gao, Xue-Li Zhao

**Affiliations:** ^1^ Key Laboratory for Forest Resources Conservation and Utilization in the Southwest Mountains of China, Ministry of Education, College of Forestry, Southwest Forestry University, Kunming, China; ^2^ School of Ecology and Environmental Science and Yunnan Key Laboratory for Plateau Mountain Ecology and Restoration of Degraded Environments, Yunnan University, Kunming, China; ^3^ Chinese Academy of Sciences (CAS) Key Laboratory of Mountain Ecological Restoration and Bioresource Utilization and Ecological Restoration and Biodiversity Conservation Key Laboratory of Sichuan Province, Chengdu Institute of Biology, Chinese Academy of Sciences, Chengdu, China

**Keywords:** *Indigofera*, plastid genome, phylogeny, positive selection, Papilionoideae, Leguminosae

## Abstract

**Introduction:**

*Indigofera* L. is the third largest genus in Fabaceae and includes economically important species that are used for indigo dye-producing, medicinal, ornamental, and soil and water conservation. The genus is taxonomically difficult due to the high level of overlap in morphological characters of interspecies, fewer reliability states for classification, and extensive adaptive evolution. Previous characteristic-based taxonomy and nuclear ITS-based phylogenies have contributed to our understanding of *Indigofera* taxonomy and evolution. However, the lack of chloroplast genomic resources limits our comprehensive understanding of the phylogenetic relationships and evolutionary processes of *Indigofera*.

**Methods:**

Here, we newly assembled 18 chloroplast genomes of *Indigofera*. We performed a series of analyses of genome structure, nucleotide diversity, phylogenetic analysis, species pairwise Ka/Ks ratios, and positive selection analysis by combining with allied species in Papilionoideae.

**Results and discussion:**

The chloroplast genomes of *Indigofera* exhibited highly conserved structures and ranged in size from 157,918 to 160,040 bp, containing 83 protein-coding genes, 37 tRNA genes, and eight rRNA genes. Thirteen highly variable regions were identified, of which *trn*K-*rbc*L, *ndh*F-*trn*L, and *ycf*1 were considered as candidate DNA barcodes for species identification of *Indigofera*. Phylogenetic analysis using maximum likelihood (ML) and Bayesian inference (BI) methods based on complete chloroplast genome and protein-coding genes (PCGs) generated a well-resolved phylogeny of *Indigofera* and allied species. *Indigofera* monophyly was strongly supported, and four monophyletic lineages (i.e., the Pantropical, East Asian, Tethyan, and Palaeotropical clades) were resolved within the genus. The species pairwise Ka/Ks ratios showed values lower than 1, and 13 genes with significant posterior probabilities for codon sites were identified in the positive selection analysis using the branch-site model, eight of which were associated with photosynthesis. Positive selection of *accD* suggested that *Indigofera* species have experienced adaptive evolution to selection pressures imposed by their herbivores and pathogens. Our study provided insight into the structural variation of chloroplast genomes, phylogenetic relationships, and adaptive evolution in *Indigofera*. These results will facilitate future studies on species identification, interspecific and intraspecific delimitation, adaptive evolution, and the phylogenetic relationships of the genus *Indigofera*.

## 1 Introduction

Fabaceae is considered the third largest family of angiosperms, which is traditionally divided into three subfamilies including Caesalpinioideae, Mimosoideae, and Papilionoideae (Lewis et al., 2005; LPWG, 2013). However, six subfamilies (i.e., Caesalpinioideae, Cercidoideae, Detarioideae, Dialioideae, Duparquetioideae, and Papilionoideae) were proposed based on a phylogenetic framework reconstructed from the chloroplast *mat*K gene (LPWG, 2017). *Indigofera* L. is the third largest genus in Fabaceae, comprising approximately 750 species with a pantropical distribution (Schrire, 2005). The genus has four centers of diversity in Africa and Madagascar (c. 550 species), Asia, especially the temperate Sino-Himalayan region (c. 105 species), Australia (c. 50 species), and the New World (c. 45 species) (Schrire et al., 2009). The genus *Indigofera* includes economically important species (Gerometta et al., 2020), used for a variety of purposes. *Indigofera tinctoria* L. and *I. suffruticosa* Mill. are the main sources for the production of natural indigo (Marquiafável et al., 2009; Baran et al., 2010; Calvo et al., 2011; Schrire, 2013; Zhang et al., 2019; Gerometta et al., 2020), which has the advantages of low toxicity and abundant availability compared to chemically synthesized dyes (Shahid-ul-Islam et al., 2013; Pattanaik et al., 2019). Moreover, indigo production is one of indispensable economic industries in India (Gulrajani, 2001; Bechtold et al., 2002). *Indigofera* also includes antiarthritic, anthelmintic, anticancer, antibacterial, and antidiabetic medicinal plants (Rajkapoor et al., 2009; Kumar et al., 2011; Dkhil et al., 2020), and a host of forage crops, ornamental, and soil conservation plants (Hassen, 2007; Marquiafável et al., 2009; Schrire, 2013). Due to the large number of species, often homogeneous morphology, overlap in the morphology of related species, and wide distribution, *Indigofera* is a taxonomically difficult genus at both the morphological and molecular levels (Schrire, 1995; Zhao, 2016). To evaluate generic relationships in the tribe Indigofereae, Barker et al. (2000) conducted the first molecular phylogenetic analyses of Indigofereae based on nuclear ITS and two chloroplast DNA (cpDNA) regions (*trn*L and *trn*K introns). Generic relationships were not well resolved because of the inconsistent phylogenetic topologies from cpDNA and ITS sequences. These early efforts suggested that these two cpDNA regions were inadequate for phylogenetic reconstruction within Indigofereae due to their low resolution. Subsequently, phylogenetic analyses of *Indigofera* have been performed using nuclear ITS sequences (Schrire et al., 2003, 2009; Zhao, 2016). Schrire et al. (2003, 2009). classified *Indigofera* into four large monophyletic clades based on a combined molecular (nuclear ITS) and morphological data set, i.e., the Pantropical, Palaeotropical, Tethyan/Boreotropical, and Cape clades. All of these studies have laid an important foundation for the taxonomy and identification of *Indigofera* species. However, the lack of cpDNA data limits our understanding of the phylogenetic relationships, biogeography, and evolutionary history of *Indigofera*. Based on these studies, Zhao (2016) conducted a large-scale phylogeny of 295 *Indigofera* species based on ITS sequences, with a focus on Sino-Himalayan endemics, and a rapid radiation in the Sino-Himalayan region was detected.

Chloroplasts play critical roles in the survival, adaptation, and evolution of plants (Wicke et al., 2011; Zhao et al., 2019; Dopp et al., 2021). Chloroplast DNA has an independent transcription and transport system and encodes ribosomal proteins related to photosynthesis (Jansen and Ruhlman, 2012). The chloroplast genome generally is a circular quadripartite structure with a length of 107 kb (*Cathaya argyrophylla* Chun & Kuang)—218 kb (*Pelargonium hortorum* L. H. Bailey) (Sato et al., 1999; Lin et al., 2010; Daniell et al., 2016), and composed of a pair of inverted repeat regions (IRs) separated by a large single-copy (LSC) region and a small single-copy (SSC) region (Kolodner and Tewari, 1979; Sugiura, 1992; Daniell et al., 2016). Given its uniparental inheritance, moderate mutation rate, and relative ease of sequencing, the chloroplast genome is widely used as an effective resource for exploring the origin and evolution of plants, understanding the phylogenetic relationships of different taxonomic categories, and identifying species (Provan et al., 2001; Bock and Knoop, 2012; Schwarz et al., 2015; Lei et al., 2016; Asaf et al., 2017a; Liang et al., 2020; Chen et al., 2022). Recent advances in next-generation sequencing (NGS) and computational resources have enabled unparalleled phylogenomic analyses (Asaf et al., 2017b; Choi et al., 2022). Complete chloroplast genome data have been sporadically applied in legume phylogenomic analyses at a range of different taxonomic levels, such as in *Campylotropis* Bunge (Feng et al., 2022b), *Sophora* L. (Liao et al., 2021), *Dalbergia* L. f. (Song et al., 2019), Millettioid/Phaseoloid clade (Oyebanji et al., 2020), Papilionoideae (Choi et al., 2022), and Fabaceae (Zhang et al., 2020b). The chloroplast genome also provides insights into other aspects of evolutionary processes, such as adaptive evolution driven by natural selection. Recent studies have detected a number of positively selected chloroplast genes associated with adaptive evolution (Xie et al., 2019; Zhao et al., 2021; Yang et al., 2022). As a large and widespread genus, *Indigofera* occupies diverse pantropical habitats, where ecology and geography uniquely shaped the *Indigofera* phylogeny (Schrire et al., 2005a, 2005b). The environment, herbivores, and pathogens have imposed strong pressures on *Indigofera* species (Schrire, 2013). However, few studies have investigated the adaptive evolution of *Indigofera*, and to date, only six complete chloroplast genomes of *Indigofera* species have been reported (Zhang et al., 2020b, 2022; Zhao et al., 2023).

In this study, we sequenced, assembled, and annotated plastomes of 17 *Indigofera* species based on a taxonomically representative sampling. By leveraging another 19 previously published cp genomes from Papilionoideae, we aimed to: (1) examine the diversity of chloroplast genomes of *Indigofera*, (2) identify promising molecular markers for future study, (3) preliminarily explore the systematic position and phylogenetic relationships of the genus *Indigofera* based on cp genomes, and (4) investigate selective or adaptive evolution in the cp genomes of *Indigofera* species.

## 2 Materials and methods

### 2.1 Taxon sampling, DNA extraction, and sequencing

**Figure 1:**
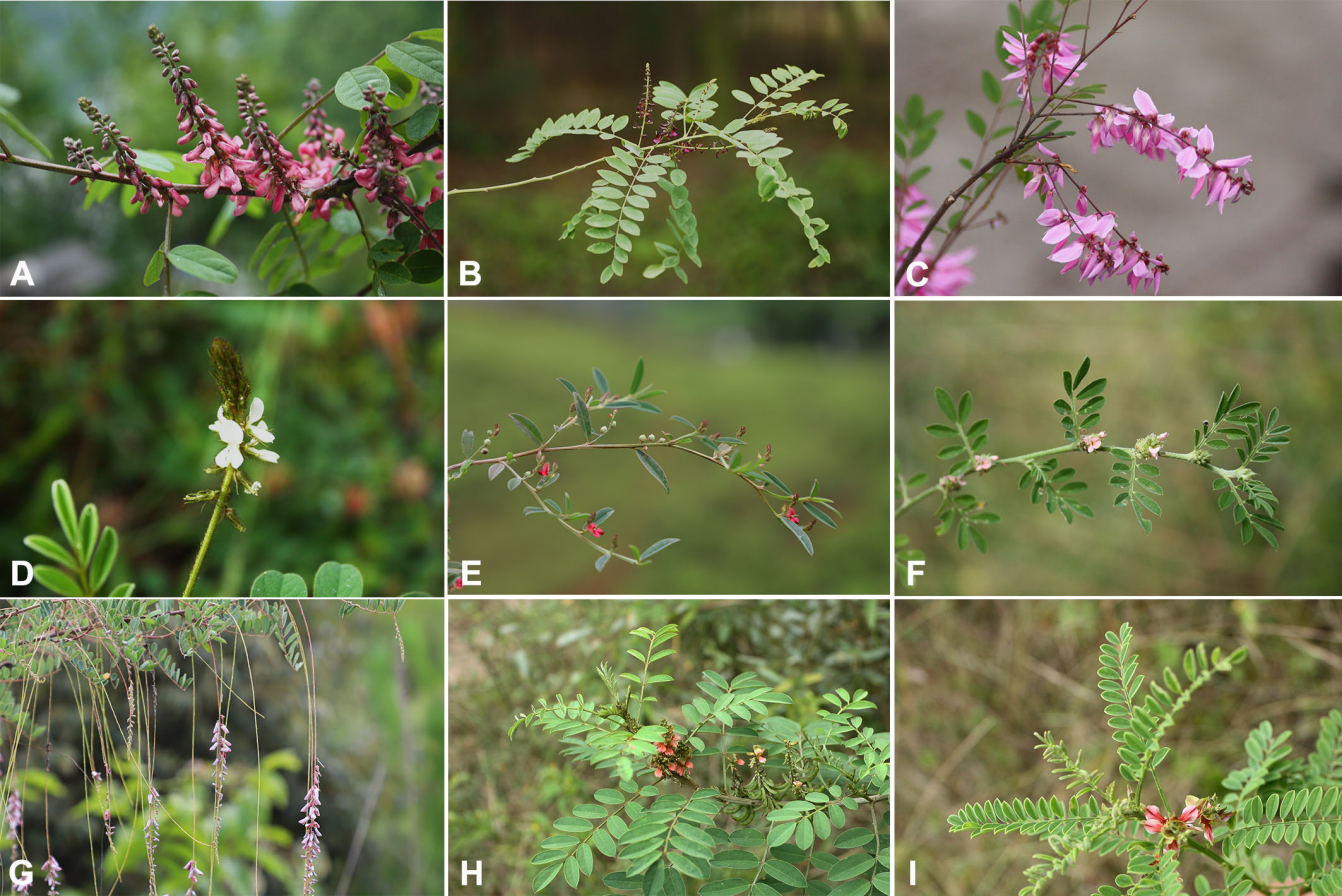
Morphological characteristics of *Indigofera* species. **(A)** *I*. *amblyantha*, **(B)** *I*. *atropurpurea*, **(C)** *I*. *decora*, **(D)** *I*. *hirsuta*, **(E)** *I*. *linifolia*, **(F)** *I*. *linnaei*, **(G)** *I*. *pendula*, **(H)** *I*. *suffruticosa*, **(I)** *I*. *wightii*.

Seventeen *Indigofera* species were selected for sampling based on their morphological characteristics and prior phylogenetic studies of *Indigofera*, which covered four ITS-based monophyletic clades (i.e., the Pantropical, Palaeotropical, Tethyan, and East Asian clades) of *Indigofera* (Schrire et al., 2003, 2009; Zhao, 2016), as well as nine morphology-based subsections (Fang and Zheng, 1994) (**Figure 1**). Fresh and healthy leaves were collected from natural populations, and then directly dried with silica gel. The voucher specimens were identified by Dr. Xue-Li Zhao and Prof. Xin-Fen Gao according to *Flora of China* (Gao and Schrire, 2010) and the speciemens of CVH (Chinese Virtual Herbarium; https://www.cvh.ac.cn/) and JSTOR (https://www.jstor.org/), and deposited at the Herbarium of Chengdu Institute of Biology, Chinese Academy of Sciences (CDBI). Voucher information and GenBank accession numbers of newly generated cp genomes are listed in **Table 1**.

**Table 1:** Characteristics of the 20 complete chloroplast genomes of *Indigofera*, including 18 newly generated plastomes and two published plastomes.

Species	Voucher No.	Size (bp)				GC content (%)	No. of genes	Accession No.	Locality
		Total	LSC	SSC	IR	Total (LSC/SSC/IR)	Total (CDS/tRNA/rRNA)		
*I. amblyantha*	ZXL36-6	159,686	89,849	19,083	25,377	35.6 (32.9/28.9/42.9)	130(83/37/8)	OQ134123	Henan, China
*I. atropurpurea*	ZZM397-10	158,847	89,437	18,868	25,271	35.8 (33.1/29.2/43.0)	129(83/37/8)	OQ147467	Guizhou, China
*I. bungeana*	ZXL97-8	159,777	90,093	19,072	25,306	35.6 (32.9/28.9/42.9)	130(83/37/8)	OQ147468	Anhui, China
*I. carlesii*	ZXL193-7	159,172	89,407	19,063	25,351	35.7 (33.1/28.9/42.9)	129(83/37/8)	OQ147469	Shanxi, China
*I. cassioides*	ZXL307	158,679	89,344	18,793	25,271	35.8 (33.1/29.2/43.0)	129(83/37/8)	OQ147470	Yunnan, China
*I. caudata*	QL2022002	158,586	89,249	18,733	25,302	35.8 (33.1/29.2/43.0)	129(83/37/8)	OQ147481	Yunnan, China
*I. decora*	ZXL112-4	159,164	89,402	19,060	25,351	35.7 (33.1/28.9/42.9)	129(83/37/8)	OQ147471	Fujian, China
*I. franchetii*	ZXL2014-22-3	158,531	89,002	18,901	25,314	35.8 (33.1/29.1/43.0)	129(83/37/8)	OQ147466	Yunnan, China
*I. hebepetala* var. *glabra*	GXF14753	158,457	89,111	18,768	25,289	35.8 (33.1/29.2/43.0)	129(83/37/8)	OQ147482	Tibet, China
*I. hirsuta*	ZXL220-3	159,873	90,361	18,858	25,327	35.5 (32.8/28.8/42.9)	129(83/37/8)	OQ147472	Hainan, China
*I. kirilowii*	ZXL166-9	159,615	89,879	19,034	25,351	35.7 (33.0/28.9/42.9)	129(83/37/8)	OQ147473	Beijing, China
I. linifolia*	15CS10171	160,040	90,459	18,935	25,323	35.8 (33.1/29.4/42.9)	130(83/37/8)	NC_047353	Yunnan, China
*I. linnaei* 1	ZXL215-11	159,455	89,892	18,943	25,310	35.7 (33.0/29.1/42.9)	130(83/37/8)	OQ147474	Hainan, China
*I. linnaei* 2	ZXL219-5	159,450	89,890	18,940	25,310	35.7 (33.0/29.1/42.9)	130(83/37/8)	OQ147475	Hainan, China
I. miniata*	C. Lee 1142 (TEX_LL)	159,935	90,400	18,897	25,319	35.8 (33.1/29.3/42.9)	128(83/37/8)	MW628948	Not available
*I. pendula*	ZXL2014-14-20	159,034	89,512	18,896	25,313	35.7 (33.0/29.1/43.0)	129(83/37/8)	OQ147476	Yunnan, China
*I. scabrida*	ZXL2014-37-2	157,918	88,490	18,870	25,279	35.9 (33.2/29.1/43.0)	129(83/37/8)	OQ147477	Ynnan, China
*I. stachyodes*	ZZM334-1	158,425	89,156	18,733	25,268	35.8 (33.1/29.2/43.0)	129(83/37/8)	OQ147478	Guizhou, China
*I. suffruticosa*	ZXL235-5	158,517	88,896	18,893	25,364	35.8 (33.1/29.2/42.9)	129(83/37/8)	OQ147479	Hainan, China
*I. wightii*	ZXL236-12	159,787	90,109	19,098	25,290	35.7 (33.0/28.9/43.0)	130(83/37/8)	OQ147480	Hainan, China

*Chloroplast genome of *Indigofera* published by GenBank.

Total genomic DNA was extracted from leaf tissues using a Plant Genomic DNA Kit (Tiangen Biotech, Beijing, China). The DNA concentration, purity, and integrity were evaluated using the Nanodrop 2000 spectrophotometry (Thermo Scientific, US) and 1% agarose gel electrophoresis. High-quality DNA samples were used to construct paired-end libraries. The paired-end libraries were subsequently sequenced on the Illumina NovaSeq 6000 platform (PE150) at Annoroad Gene Technology Co., Ltd (Beijing, China).

### 2.2 Chloroplast genome assembly and annotation

The raw data were trimmed to remove adapters and low-quality reads using Trimmomatic v.0.39 (Bolger et al., 2014). The clean reads were then assembled using GetOrganelle v1.7.6.1 (Jin et al., 2020) with the k-mers of 21, 45, 65, 85, 105, and 121. *De novo* assembly graphs were visualized using Bandage v0.8.1 (Wick et al., 2015). Geneious Prime 2022.1.1 (Biomatters, Auckland, New Zealand) was used to identify the location of IRs. Gene annotation was conducted using Plastid Genome Annotator (Qu et al., 2019) with a published chloroplast genome of *Indigofera linifolia* (L. f.) Retz. (NC_047353) as a reference, and the start/stop codons and pseudogenes were further manually checked. The physical chloroplast genome maps were drawn using OGDRAW v1.3.1 (Greiner et al., 2019).

### 2.3 Contraction and expansion of the IRs and identification of polymorphic regions

A total of 18 newly assembled and two published *Indigofera* cp genomes (*I. linifolia* and *I. miniata* Ort.) (**Table 1**) were analyzed. Sequences were aligned using the program MAFFT v7 (Katoh and Standley, 2013) with default parameters. The boundaries of LSC, SSC, and IRs in the cp genomes were identified and visualized using IRscope (Amiryousefi et al., 2018). The GC content, gene components, and length of the whole cp genome, LSC, SSC, and IRs were analyzed using Geneious Prime 2022.1.1 (Biomatters Ltd. Auckland, New Zealand). Variable and parsimony-informative sites were detected by MEGA v11.0.11 (Tamura et al., 2021). Nucleotide diversity (*Pi*) was calculated using DnaSP v6.12.03 (Rozas et al., 2017) with a window length of 600 bp and step size of 200 bp.

### 2.4 Repeat sequence analysis

Simple sequence repeats (SSRs) were detected using MISA v2.1 (Beier et al., 2017). Thresholds of 11, 6, 5, 4, 3, and 3 repeat units were set for mono-, di-, tri-, tetra-, penta-, and hexa-nucleotides, respectively. Long repeat sequences (forward, reverse, palindromic, and complementary) were identified using REPuter (Kurtz et al., 2001), with a minimum repeat size of 30 bp and a Hamming distance of 3.

### 2.5 Phylogenetic analysis

To investigate the systematic placement and phylogenetic relationships of the *Indigofera* species, 19 publicly available cp genome sequences in Papilionoideae were downloaded from NCBI (**Table 1** and **Supplementary Table S1**). A total of 37 cp genomes were used to construct phylogenetic relationships. Phylogenetic analyses were conducted using maximum likelihood (ML) and Bayesian inference (BI) methods based on two data sets: once using the complete plastome, and once constraining analysis to the concatenated protein-coding genes (PCGs). The PCGs were extracted from each cp genome, then concatenated and aligned with PhyloSuite v1.2.2 (Zhang et al., 2020a). Multiple sequence alignment was aligned by MAFFT v7 (Katoh and Standley, 2013), and then trimmed using trimAL v1.2 (Capella-Gutiérrez et al., 2009) with automatic mode to reduce potentially poorly aligned regions. The trimmed alignment was visually examined and manually adjusted in Geneious Prime 2022.1.1 (Biomatters Ltd. Auckland, New Zealand).

The best-fit models of nucleotide substitution were estimated by jModelTest v2.1.10 (Guindon and Gascuel, 2003; Darriba et al., 2012) using a corrected Akaike Information Criterion (AIC) score (Akaike, 1974). ML analyses were implemented using IQ-TREE v2.2.0 (Minh et al., 2020) under the GTR+I+J model with 1,000 bootstrap replicates. Bayes inference was performed using MrBayes v3.2.7 (Ronquist et al., 2012) under the GTR+I+G model. Markov Chain Monte Carlo (MCMC) algorithms have two parallel runs with 10,000,000 generations independently, and sampling every 1,000 generations. The initial 25% of trees were discarded as burn-in, and the remaining trees were used to construct a 50% majority-rule consensus tree and calculate the posterior probability (PP) values using Tracer v1.7.1 (Rambaut et al., 2018). The phylogenetic trees were visualized and processed using Interactive Tree Of Life (iTOL) v5 (Letunic and Bork, 2021) and Figtree v1.4.4 (https://github.com/rambaut/figtree/releases/tag/v1.4.4).

### 2.6 Species pairwise Ka/Ks ratios and positive selected analyses

Each shared PCG sequence of all species was extracted and aligned with MAFFT v7 (Katoh and Standley, 2013). Pairwise Ka/Ks of all species were calculated based on the concatenated PCGs alignment using DnaSP v6.12.03 (Rozas et al., 2017).

Positive selected analyses of shared genes were performed with the branch-site model (Yang and Reis, 2011) and Bayesian Empirical Bayes (BEB) methods (Yang et al., 2005). A total of 70 shared PCGs of all species were extracted and aligned with MACSE v2 (Ranwez et al., 2018). Before calculation, stop codons and gaps were removed. The branch-site model was implemented to assess potential positive selection in the CODEML algorithm from the PAML v4.10.6 package (Yang, 1997, 2007). A null hypothesis (model = 2, NSsites = 2, omega = 1, fix_omega = 1) and an alternative hypothesis (model = 2, NSsites = 2, omega = 0, fix_omega = 1.5) models were applied separately. The ratio (ω) of the non-synonymous substitution rate to the synonymous substitution rate was used to measure the selective pressure. Whether ω > 1, ω = 1, or ω < 1 indicates positive selection, neutral evolution, and purifying selection, respectively (Yang and Nielsen, 2002). The likelihood-ratio tests (LRT) were performed according to Lan et al. (2017). The posterior probabilities of amino acid sites were calculated with the BEB method to identify specific positions experiencing positive selection (Yang et al., 2005). A gene with a test *p*-value < 0.05 at a site associated with positive selection was thus considered a positively selected gene (Xie et al., 2019).

## 3 Results

### 3.1 Chloroplast genome features of *Indigofera*

**Figure 2:**
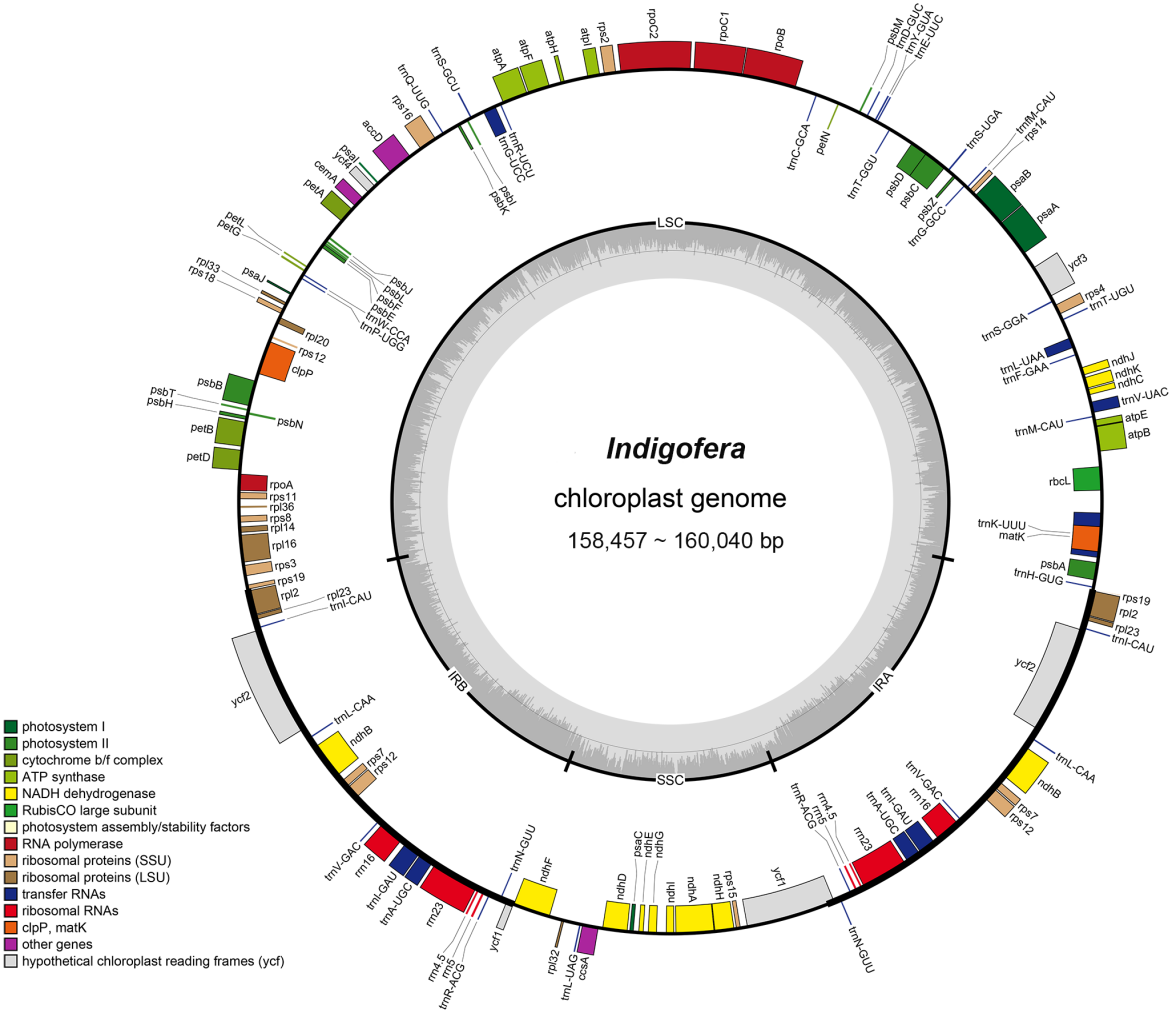
The chloroplast genome map of *Indigofera* species. Genes inside and outside of the circle are transcribed clockwise and counterclockwise, respectively. Genes belonging to different functional groups are color-coded. The darker and lighter gray color in the inner circle corresponds to the GC content and AT content, respectively.

Complete chloroplast genomes of 17 *Indigofera* species were newly sequenced and annotated in the present study, of which four species (*I. pendula* Franch., *I. franchetii* X. F. Gao & Schrire, *I. amblyantha* Craib and *I. carlesii* Craib) are endemic to China. We studied the basic information of plastomes of 17 species of *Indigofera* using the published sequences from *I. linifolia* and *I. miniata* in GenBank. Illumina sequencing generated about 3 Gb of paired-end raw sequence data for each newly sampled *Indigofera* species. The complete cp genome ranged from 157,918 bp (*I. scabrida* Dunn) to 160,040 bp (*I. linifolia*). Every species exhibited a typical quadripartite genome structure, consisting of an LSC region (88,490—90,459 bp) and an SSC region (18,733—19,098 bp) separated by a pair of inverted repeats (25,268—25,377 bp) (**Table 1** and **Figure 2**). The GC content of these complete cp genomes ranged from 35.5% (*I. hirsuta* L.) to 35.9% (*I. scabrida*), in which the IR regions possessed the highest GC content (42.9%—43%), followed by the LSC (32.8%—33.2%), and SSC regions (28.8%—29.4%). The annotated cp genomes of *Indigofera* contained a total of 128—130 genes, including 83 protein-coding genes (10 in the IR regions), 37 tRNA genes (14 in the IR regions), and eight rRNA genes (all in the IR region) (**Table 1** and **Figure 2**). Of these genes, four tRNA genes (*trn*G*-*UCC, *trn*K*-*UUU, *trn*L*-*UAA, and *trn*V*-*UAC) contained one intron, and two tRNA genes (*trn*A*-*UGC and *trn*I*-*GAU) contained two introns (**Table 2**). Nineteen genes contained two copies, including eight protein-coding genes (*ndh*B, *rpl*2, *rpl*23, *rps*7, *rps*12, *rps*19, *ycf*1, and *ycf*2), four rRNA genes (*rrn*16, *rrn*23, *rrn*4.5, and *rrn*5), and seven tRNA genes (*trn*A-UGC, *trn*I-CAU, *trn*I-GAU, *trn*L-CAA, *trn*N-GUU, *trn*R-ACG, and *trn*V-GAC). In these cp genomes, *rps*12 was identified as a trans-spliced gene, with the 5’-end in the LSC region, and the 3’-end in the IR regions.

**Table 2:** Summary of gene contents present in the *Indigofera* chloroplast genomes.

Gene group	Gene
Subunits of photosystem I	*psa*A, *psa*B, *psa*C, *psa*I, *psa*J
Submits of photosystem II	*psb*A, *psb*B*, psb*C, *psb*D, *psb*E, *psb*F, *psb*H, *psb*I, *psb*J, *psb*K*, psb*L, *psb*M, *psb*N, *psb*T, *psb*Z
Subunits of NADH dehydrogenase	*ndh*A, *ndh*B(×2), *ndh*C, *ndh*D, *ndh*E, *ndh*F, *ndh*G, *ndh*H, *ndh*I, *ndh*J, *ndh*K
Subunits of cytochrome b/f complex	*pet*A, *pet*B, *pet*D, *pet*G, *pet*L, *pet*N
Subunits of ATP synthase	*atp*A, *atp*B, *atp*E, *atp*F, *atp*H, *atp*I
Large subunit of RuBisco	*rbc*L
Proteins of large ribosomal subunit	*rpl*2(×2)*, rpl*14*, rpl*16*, rpl*20*, rpl*23(×2)*, rpl*32*, rpl*33*, rpl*36
Proteins of small ribosomal subunit	*rps*2, *rps*3, *rps*4, *rps*7(×2), *rps*8, *rps*11, *rps*12(×2), *rps*14, *rps*15, *rps*16, *rps*18, *rps*19(×2)
Subunits of RNA polymerase	*rpo*A, *rpo*B, *rpo*C1, *rpo*C2
Ribosomal RNAs	*rrn*16(×2), *rrn*23(×2), *rrn*4.5(×2), *rrn*5(×2)
Transfer RNAs	*trn*A-UGC**(×2), *trn*C-GCA, *trn*D-GUC, *trn*E-UUC, *trn*F-GAA, *trn*fM-CAU, *trn*G-UCC*, *trn*G-GCC, *trn*H-GUG, *trn*I-CAU(×2), *trn*I-GAU**(×2), *trn*K-UUU*, *trn*L-UAA*, *trn*Y-GUA, *trn*L-CAA(×2), *trn*L-UAG, *trn*M-CAU, *trn*N-GUU(×2), *trn*P-UGG, *trn*Q-UUG, *trn*R-ACG(×2), *trn*R-UCU, *trn*S-GCU, *trn*S-UGA, *trn*S-GGA, *trn*T-CGU, *trn*T-UGU, *trn*V-GAC(×2), *trn*V-UAC*, *trn*W-CCA
Maturase	*mat*K
Protease	*clp*P
Envelope membrane protein	*cem*A
Acetyl-CoA carboxylase	*acc*D
Cytochrome c biogenesis	*ccs*A
Conserved open reading frames	*ycf*1(×2), *ycf*2(×2), *ycf*3, *ycf*4

*Genes with one intron; **Genes with two introns; (×2) Genes with two copies.

### 3.2 Comparative analysis of IR boundaries

We observed both contraction and expansion of IRs at the SC-IR boundary. Among these 20 cp genomes, the IRs regions varied from 25,268 to 25,377 bp (**Table 1**). The *rps*19 and *rpl*2 were located at the JLB (LSC/IRb) boundary, and the *ycf*1 pseudogene (except for *I. miniata*) and *ndh*F at the JSB (SSC/IRb) boundary (**Supplementary Figure S1**). At the JLB boundary, the *rps*19 region in *I. amblyantha*, *I. bungeana* Walp., *I. linifolia*, *I. linnaei* Ali, *I. miniata*, and *I. wightii* Graham expanded 2—6 bp toward the IRb region and led to the production of *rps*19 pseudogene in the IRa (except in the case of *I. miniata*). The *ycf*1 crossed the JSA (SSC/IRa) boundary with 4,881—4,920 bp in the SSC region and expanded from 456 to 478 bp into the IRa region, which led to the *ycf*1 pseudogene (except for *I. miniata*). At the JSB boundary, the *ndh*F region of *I. amblyantha*, *I. atropurpurea* Bench.-Ham. ex Hornem., *I. bungeana*, *I. cassioides* Rottler ex Candolle, *I. hebepetala* var. *glabra* Ali, *I. hirsuta*, *I. linifolia*, *I. stachyodes* Lindl., *I. suffruticosa*, and *I. wightii* expanded 3—12 bp toward IRb.

### 3.3 Divergence hotspot regions

**Figure 3:**
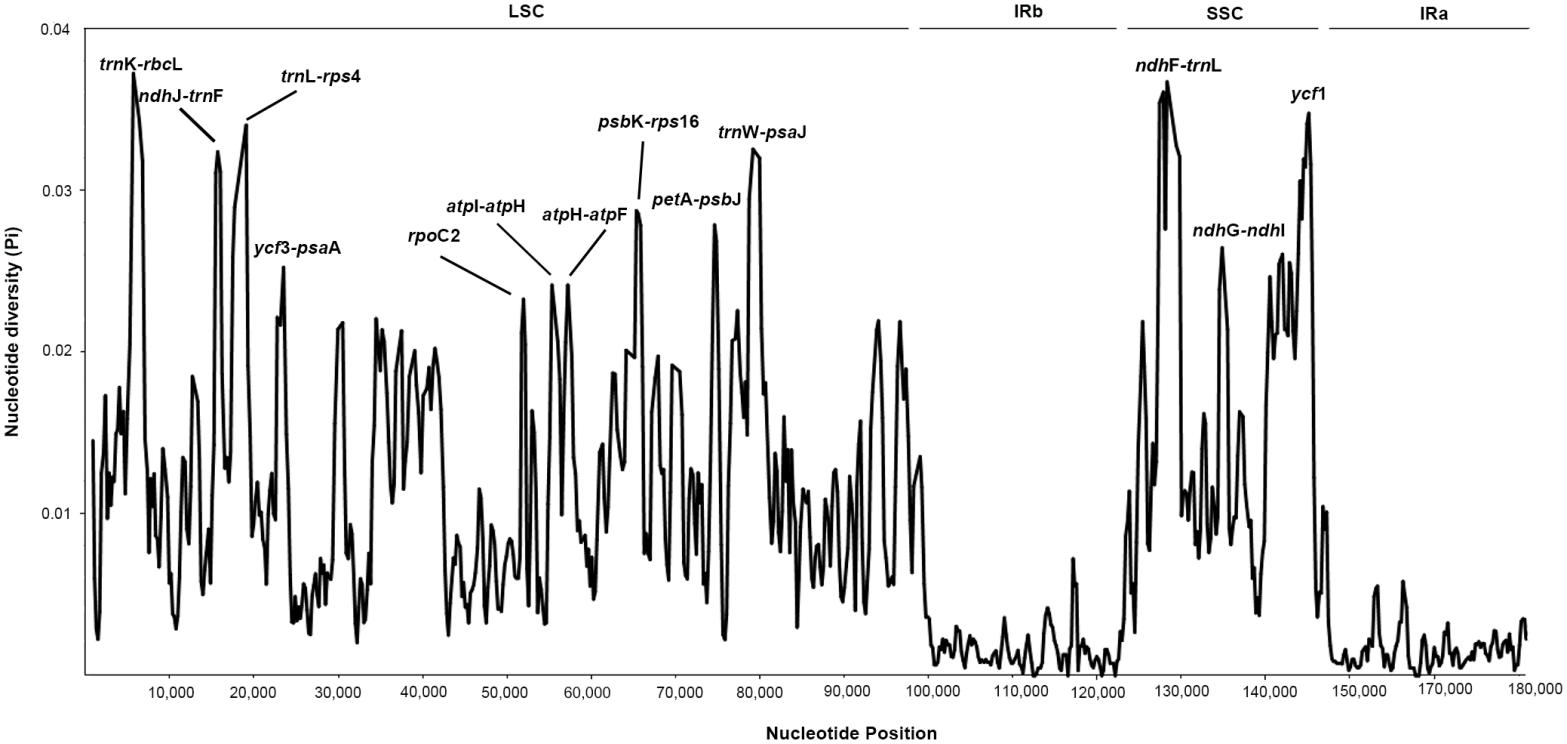
The nucleotide diversity (*Pi*) value (Y-axis) with their positions (X-axis) in each window of the *Indigofera* species chloroplast genomes based on sliding window analysis. The 13 regions with high diversity are indicated above the peaks.

To explore variable regions with high resolution for species identification in *Indigofera*, we conducted a sliding windows analysis of the aligned sequences of 20 cp genomes. The sliding windows analysis observed 13 highly variable regions, comprising the intergenic spacer regions and introns: *trn*K*-rbc*L (1,542 bp), *ndh*J*-trn*F (761 bp), *trn*L*-rps*4 (1,846 bp), *ycf*3*-psa*A (1,075 bp), *rpo*C2 (621 bp), *atp*I*-atp*H (1,163 bp), *atp*H*-atp*F (983 bp), *psb*K*-rps*16 (734 bp), *pet*A*-psb*J (748 bp), *trn*W*-psa*J (1,449 bp), *ndh*F*-trn*L (1,699 bp), *ndh*G*-ndh*I (1,153 bp), and *ycf*1 (627 bp) (**Table 3** and **Figure 3**). The 13 highly variable regions had remarkably higher *Pi* values (0.02329 to 0.03725), and the largest *Pi* value was for *trn*K-*rbc*L (0.03725). All these highly variable regions were located in the LSC and SSC regions (**Figure 3** and **Supplementary Table S2**).

**Table 3:** Polymorphic regions among the 20 cp genomes of *Indigofera*.

Gene	Start	End	Length (bp)	Nucleotide diversity (*Pi*)	No. singleton variable sites (%)	No. parsimony information sites (%)
*trn*K*-rbc*L	5,063	6,604	1,542	0.03725	119 (7.72%)	129 (8.37%)
*ndh*J*-trn*F	15,343	16,103	761	0.03241	39 (5.12%)	51 (6.70%)
*trn*L*-rps*4	17,581	19,426	1,846	0.03406	127 (6.88%)	146 (7.91%)
*ycf*3*-psa*A	22,873	23,947	1,075	0.02525	76 (7.07%)	62 (5.77%)
*rpo*C2	51,651	52,271	621	0.02329	21 (3.38%)	34 (5.48%)
*atp*I*-atp*H	54,973	56,135	1,163	0.02416	42 (3.61%)	75 (6.45%)
*atp*H*-atp*F	56,815	57,797	983	0.02416	48 (4.88%)	85 (8.65%)
*psb*K*-rps*16	65,248	65,981	734	0.02859	34 4.63%)	55 (7.49%)
*pet*A*-psb*J	74,271	75,018	748	0.02788	43 (5.75%)	51 (6.82%)
*trn*W*-psa*J	78,633	80,081	1,449	0.03257	105 (7.25%)	167 (11.53%)
*ndh*F*-trn*L	127,976	129,674	1,699	0.03673	136 (8.00%)	172 (10.12%)
*ndh*G*-ndh*I	134,427	135,579	1,153	0.02647	73 (6.33%)	93 (8.07%)
*ycf*1	144,818	145,444	627	0.03479	50 (7.97%)	55 (8.77%)

### 3.4 Analyses of SSR and long repeat sequence

**Figure 4:**
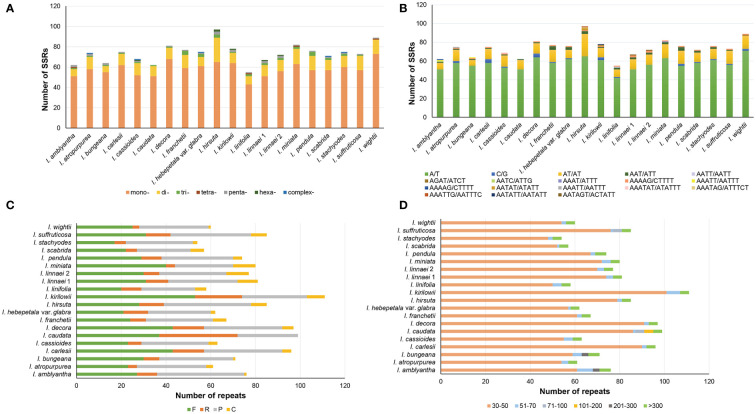
Analysis of SSRs and long sequence repeat in each species. **(A)** The number of different SSR types. **(B)** The number of the identified SSRs in different repeat class types. **(C)** The type and number of long repeat sequences. **(D)** Length distribution of long repeat sequences.

The number of SSRs in 20 *Indigofera* plastomes varied from 55 in *I. linifolia* to 97 in *I. hirsuta*. The most abundant SSRs were mononucleotide repeats, where the number varied from 43 to 73, followed by dinucleotide repeats (6—24) (**Figure 4A** and **Supplementary Table S3**). Moreover, 94.03%—100% of SSRs were distributed in the LSC and SSC regions (**Supplementary Table S4**). Mononucleotides and dinucleotides were found in all species of *Indigofera* and trinucleotides and pentanucleotides were present in all species except *I. wightii* or *I. caudata*. Tetranucleotides, hexanucleotides, and complex nucleotides were detected in some species (**Figure 4A** and **Supplementary Table S3**). We found that SSRs in the plastomes of *Indigofera* contained a large number of A/T pairs and AT/AT repeats (**Figure 4B** and **Supplementary Table S5**).

A total of 1,514 repeat sequences were identified in 20 *Indigofera* plastomes using REPuter, including 597 forward (39.43%), 202 reverse (13.34%), 620 palindromic (40.95%), and 95 complementary (6.27%) repeats (**Figure 4C** and **Supplementary Table S6**). The proportion of long repeats detected in the LSC, SSC, IRa, and IRb regions was 25.25%—77.59%, 3.33%—52.53%, 1.72%—7.06%, and 12.07%—25%, respectively (**Supplementary Table S7**). Repeat lengths less than 100 bp were the most common, accounting for 88.73%—96.40% of total repeats. Among these, long repeat lengths of 30—50bp were the most common. Repeat lengths of 101—200 bp were only observed in *I. caudata*, and 201—300 bp repeats only in *I. amblyantha* and *I. bungeana*. However, repeat lengths greater than 300 bp were detected in all species (**Figure 4D** and **Supplementary Table S8**).

### 3.5 Phylogenetic relationships

**Figure 5:**
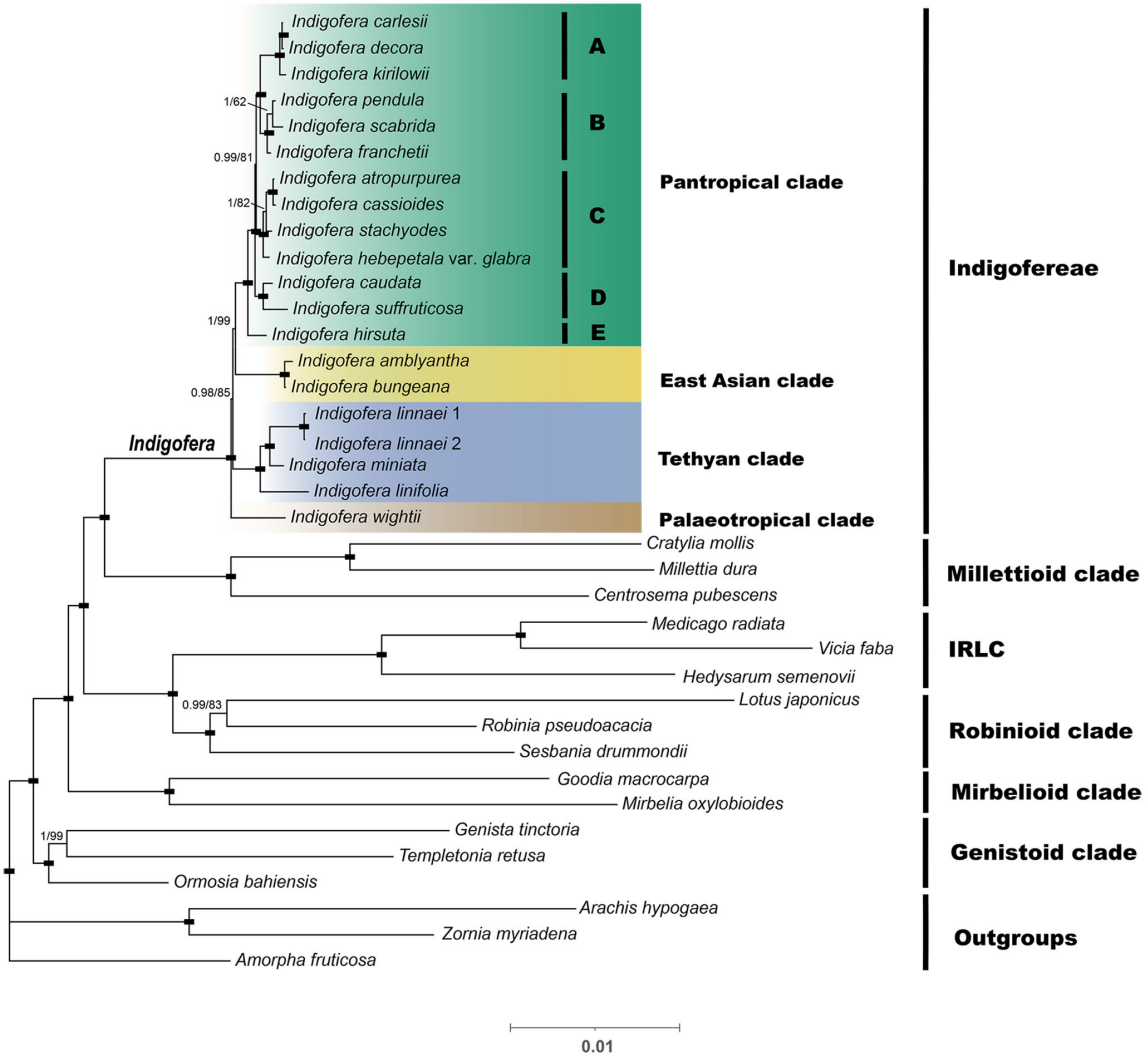
A Bayesian inference (BI) tree based on a concatenated dataset of protein-coding genes (PCGs). The numbers above the branches are Bayesian posterior probabilities (PP; before the slash) and ML bootstrap supports values (BS; after the slash). The black rectangle on the node indicates support for BS=100 and PP=1. The paleotropical clade includes five lineages, i.e., A-E.

The data matrix of the complete genome used in phylogenetic analyses consisted of 129,037 nucleotide sites, of those, 34,002 (26.35%) were parsimony informative. The PCG data matrix consisted of 69,282 nucleotide sites, of these, 8,906 (12.85%) were parsimony informative. The ML and BI analyses generated identical tree topologies of *Indigofera*, whereas the topological discrepancies were observed in the Robinioid and Genistoid clades among phylogenies from two datasets (**Figure 5** and **Supplementary Figures S2, S3**).

The plastid phylogenomic analyses generated highly supported phylogeny with six monophyletic clades, i.e., Millettioid, IRLC, Robinioid, Mirbelioid, Genistoid, and *Indigofera* clades, with the Millettioid clade being the most closely related to *Indigofera*. All phylogenetic topologies fully supported a monophyletic *Indigofera* [100% bootstrap support (BS) and 1 posterior probability (PP); **Figure 5** and **Supplementary Figures S2, S3**]. Nineteen *Indigofera* species were resolved into four monophyletic clades, namely Pantropical, East Asian, Tethyan, and Palaeotropical clades according to Schrire et al. (2009) and Zhao (2016). Within the Pantropical clade, 13 species formed five monophyletic lineages (**Figure 5A-E**). The East Asian clade was composed of *I. amblyantha* and *I. bungeana*. Within the Tethyan clade, *I. linnaei* was sister to *I. miniata* and related to *I. linifolia*. The Palaeotropical clade included only *I. wightii*. In contrast to the PCGs phylogenetic trees, the ML and BI trees based on the complete cp genome suggested *I. pendula* is a highly supported sister to *I. franchetii* (100% BS, 1 PP) in the Pantropical clade. Moreover, our analyses suggested the Tethyan clade was moderately (85%/66% BS, 0.98/0.99 PP) supported as a sister clade of the Pantropical and East Asian clades (**Figure 5** and **Supplementary Figures S2, S3**).

### 3.6 The pairwise Ka/Ks ratios and positive selection analyses

**Figure 6:**
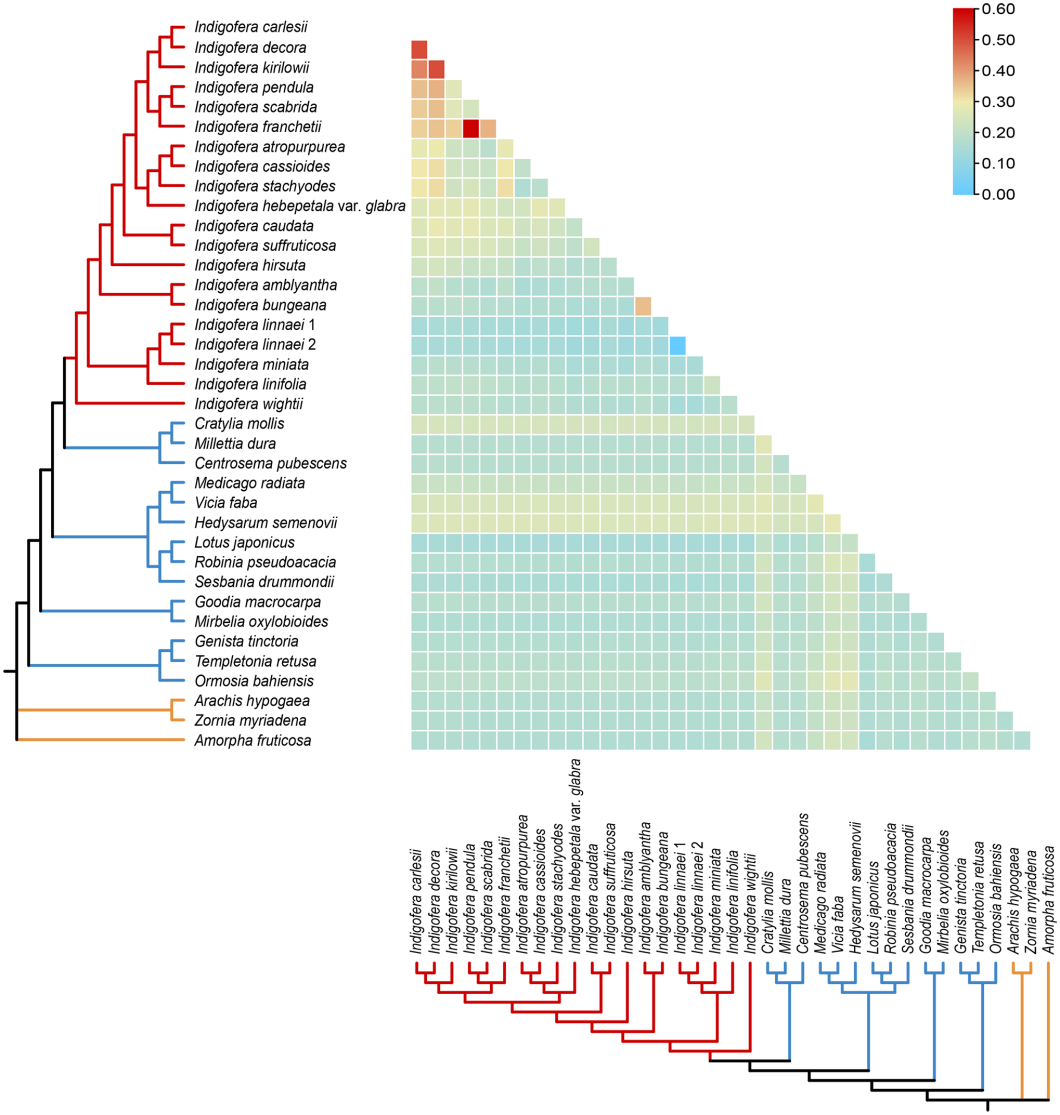
Pairwise Ka/Ks ratios in *Indigofera* and allied species. This heatmap shows pairwise Ka/Ks ratios between every concatenated shared PCGs sequence in the multigene nucleotide alignment. The scale factors associated with each value are shown on the top right side of the figure.

The pairwise Ka/Ks ratios of each species pair were calculated, and the ratios ranged from 0 to 0.6 (**Figure 6** and **Supplementary Table S9**). Higher pairwise Ka/Ks ratios were observed in *Indigofera* species pairs than non-*Indigofera* species pairs. In addition, high Ka/Ks ratios were detected in the species pairs associated with the Pantropical clade, such as *I. franchetii vs*. *I. pendula*, *I. decora* Lindl. *vs*. *I. carlesii*, *I. kirilowii* Maxim. ex Palibin *vs*. *I. decora* and *I. kirilowii vs*. *I. carlesii*.

A total of 70 PCGs were used for positive selection analysis with the branch-site model (**Table 4**). No *p*-values were significant in each gene range by the likelihood ratio test, however, 13 PCGs (*acc*D, *ccs*A, *mat*K, *ndh*A, *ndh*E, *ndh*F, *ndh*I, *pet*A, *pet*N, *psa*A, *psa*B, *rpo*B, and *rpo*C1) showed significant posterior probabilities, indicating sites with positive selection based on the BEB test. Notably, the posterior probability of the amino acid residues of *pet*N was as high as 0.968. Among these genes, most genes only had one positive selective site, whereas *acc*D, *ccs*A, *mat*K, and *psa*A have two positive selective sites.

**Table 4:** The potential positive selection test based on the branch-site model.

gene	Null hypothesis			Alternative hypothesis			Significance test	
	df	lnL	omega (ω=1)	df	lnL	omega (ω>1)	BEB	*p*-value
***acc*D**	75	-6431.814264	1	76	-6431.82067	1	84, Y, 0.789; 354, P, 0.800	9.10E-01
*atp*A	75	-5997.309967	1	76	-5997.309966	1		9.99E-01
*atp*B	75	-5824.546279	1	76	-5824.546279	1		1.00E+00
*atp*E	75	-1793.372592	1	76	-1793.372592	1		1.00E+00
*atp*F	75	-2670.995987	1	76	-2670.995986	1		9.99E-01
*atp*H	75	-890.901159	1	76	-890.901218	3.34679		9.91E-01
*atp*I	75	-2720.597774	1	76	-2720.597774	1		1.00E+00
***ccs*A**	75	-5113.773949	1	76	-5113.71246	3.28153	86, H, 0.516; 146, A, 0.562	7.26E-01
*cem*A	75	-3464.473998	1	76	-3464.473998	1		1.00E+00
*clp*P	75	-3695.647806	1	76	-3695.647806	1		1.00E+00
***mat*K**	75	-9802.115823	1	76	-9802.115823	1	68, N, 0.776; 402, L, 0.757	1.00E+00
***ndh*A**	75	-4744.36046	1	76	-4744.339358	3.13682	13, S, 0.667	8.37E-01
*ndh*C	75	-1446.353262	1	76	-1446.353262	1		1.00E+00
***ndh*E**	75	-1126.301925	1	76	-1126.301925	1	69, I, 0.804	1.00E+00
***ndh*F**	75	-13668.969054	1	76	-13668.967949	1.33062	129, S, 0.717	9.63E-01
*ndh*G	75	-2244.124624	1	76	-2244.124624	1		1.00E+00
*ndh*H	75	-4750.154437	1	76	-4750.154437	1		1.00E+00
***ndh*I**	75	-1723.455038	1	76	-1723.288485	62.57445	47, L, 0.587	5.64E-01
*ndh*J	75	-1809.523996	1	76	-1809.523983	1		9.96E-01
***pet*A**	75	-4203.297008	1	76	-4203.297008	1	196, P, 0.748	1.00E+00
*pet*B	75	-2289.765157	1	76	-2289.765157	1		1.00E+00
*pet*D	75	-1725.913319	1	76	-1725.913319	1		1.00E+00
*pet*G	75	-393.301114	1	76	-393.301091	5.81872		9.95E-01
*pet*L	75	-434.334927	1	76	-434.334927	1.34096		1.00E+00
***pet*N**	75	-286.087155	1	76	-285.811478	999	16, T, 0.968	4.58E-01
***psa*A**	75	-7608.377153	1	76	-7608.101969	47.84531	200, A, 0.558; 215, V, 0.824	4.58E-01
***psa*B**	75	-7415.976741	1	76	-7415.960046	4.79925	283, L, 0.517	8.55E-01
*psa*C	75	-749.023872	1	76	-749.023765	3.24431		9.88E-01
*psa*I	75	-414.261192	1	76	-414.261192	1		1.00E+00
*psa*J	75	-535.817111	1	76	-535.817111	1		1.00E+00
*psb*A	75	-3078.54617	1	76	-3078.54617	1		1.00E+00
*psb*B	75	-5147.761057	1	76	-5147.761057	1		1.00E+00
*psb*C	75	-4617.48408	1	76	-4617.484042	1		9.93E-01
*psb*D	75	-3074.576325	1	76	-3074.576325	1		1.00E+00
*psb*E	75	-688.055826	1	76	-688.055854	1		9.94E-01
*psb*F	75	-318.323808	1	76	-318.323808	2.2628		1.00E+00
*psb*H	75	-871.181742	1	76	-871.18174	1		9.98E-01
*psb*I	75	-379.276098	1	76	-379.276098	3.31478		1.00E+00
*psb*J	75	-349.235376	1	76	-349.235369	6.57949		9.97E-01
*psb*K	75	-776.710115	1	76	-776.710099	1		9.95E-01
*psb*L	75	-264.558583	1	76	-264.558583	1		1.00E+00
*psb*M	75	-386.351825	1	76	-386.351825	1		1.00E+00
*psb*N	75	-332.940369	1	76	-332.940369	3.3186		1.00E+00
*psb*T	75	-268.727919	1	76	-268.727919	1		1.00E+00
*psb*Z	75	-621.222348	1	76	-621.222363	1		9.96E-01
*rbc*L	75	-5737.126681	1	76	-5737.126681	1		1.00E+00
*rpl*2	75	-2295.402317	1	76	-2295.402327	3.31727		9.96E-01
*rpl*14	75	-1543.569464	1	76	-1543.569464	1		1.00E+00
*rpl*16	75	-1748.246762	1	76	-1748.246761	1		9.99E-01
*rpl*20	75	-2090.574884	1	76	-2091.118292	1		2.97E-01
*rpl*23	75	-367.838712	1	76	-367.838711	1		9.99E-01
*rpl*32	75	-1058.148523	1	76	-1058.148523	1		1.00E+00
*rpl*33	75	-1077.710102	1	76	-1077.710102	1		1.00E+00
*rpl*36	75	-503.752941	1	76	-503.752942	1		9.99E-01
*rpo*A	75	-4667.494071	1	76	-4666.461873	1		1.51E-01
***rpo*B**	75	-14126.484738	1	76	-14126.341251	6.88495	490, G, 0.797	9.99E-01
***rpo*C1**	75	-9255.62478	1	76	-9255.424137	34.53705	112, P, 0.672	5.26E-01
*rpo*C2	75	-22858.350779	1	76	-22858.350771	1		9.97E-01
*rps*2	75	-3063.281898	1	76	-3063.281898	1		1.00E+00
*rps*3	75	-3211.805038	1	76	-3211.805038	1		1.00E+00
*rps*4	75	-2476.84109	1	76	-2476.84109	1		1.00E+00
*rps*7	75	-1163.823135	1	76	-1163.823075	3.31899		9.91E-01
*rps*8	75	-2069.629651	1	76	-2069.629651	1		1.00E+00
*rps*11	75	-2025.29131	1	76	-2025.29131	1		1.00E+00
*rps*14	75	-1315.485389	1	76	-1315.485428	1		9.93E-01
*rps*15	75	-1484.88425	1	76	-1485.258033	1		3.87E-01
*rps*18	75	-1046.642715	1	76	-1046.642773	3.31848		9.91E-01
*ycf*1	75	-2826.348487	1	76	-2826.348487	1		1.00E+00
*ycf*2	75	-18613.096077	1	76	-18613.096142	3.31722		9.91E-01
*ycf*3	75	-1656.524153	1	76	-1656.313059	999		5.16E-01

Bold types are genes with positively selected sites. BEB, Bayesian Empirical Bayes.

## 4 Discussion

### 4.1 Characteristics of the chloroplast genomes of *Indigofera*

Comparative analysis of the 20 *Indigofera* cp genomes showed highly conserved genes and structures. The genome size, gene content, and gene organization of *Indigofera* varied little and GC content was consistent with observations in other legume taxa (**Table 1**) (Keller et al., 2017; Liao et al., 2021; Feng et al., 2022b). Like most angiosperms, the cp genomes of *Indigofera* exhibited the typical quadripartite structure, consisting of the LSC, SSC, IRa, and IRb regions (**Figure 2**). IR regions were more conserved compared with single-copy regions, consistent with most higher plants, something largely caused by repeated corrections caused by gene transformations between the two IR regions (Zhang et al., 2016).

The contraction and expansion of the IR regions has been proposed as an important source of size variation in cp genomes (Aii et al., 1997; Huang et al., 2014). However, in our present study, all taxa exhibited a highly conserved pattern of IR boundaries with only slight structural variations. Consistent with other research, we suggested that contraction and expansion of the IR regions can lead to the creation of pseudogenes (Raman et al., 2017; Gao et al., 2018; Ruang-Areerate et al., 2021; Han et al., 2022; Lian et al., 2022; Li et al., 2022c). A comparison of 20 *Indigofera* plastomes revealed that *rp*s19, *ycf*1, and *ndh*F demonstrated pronounced expansion or contraction, as reported in *Salvia* L., *Amygdalus* L., *Iris* L., *Prunus* L. subg. *Cerasus* Mill., and Asteraceae (Du et al., 2021; Pascual-Diaz et al., 2021; Du et al., 2022; Feng et al., 2022a; Li et al., 2022c). These subtle differences in boundaries could potentially be used for species identification.

### 4.2 Identification of candidate molecular markers

The chloroplast genome has a relatively conserved structure and moderate evolutionary rate. It is also inherited uniparentally and thus less affected by paralogous genes when constructing phylogenetic relationships (Wolfe et al., 1987; Jansen and Ruhlman, 2012; Gitzendanner et al., 2018). Because of these reasons, the cp genome has often been used to construct phylogenies (Aecyo et al., 2021; Yang et al., 2021; Chen et al., 2022; Tong et al., 2022). DNA barcodes based on cp genome screening have been widely applied for species identification, wide-range phylogenetic analyses, and population genetics, such as *rbc*L, *mat*K, *rpo*C1, *atp*F-*atp*H, *psb*K-*psb*I, *rpo*B, and *trn*H-*psb*A (Kress et al., 2005; Newmaster et al., 2006; Chase et al., 2007; Hollingsworth et al., 2011; Dong et al., 2012; Li et al., 2021; Chen et al., 2022).

Previous studies of *Indigofera* and its allied genera mainly include five plastid regions, comprising three intergenic regions of *ndh*J-*trn*F, *atp*H-*atp*F, *trn*D-*trn*T, and two introns of *trn*L and *mat*K (Barker et al., 2000; Peng et al., 2015; Zhao and Gao, 2015; Zhao et al., 2017). Among these hypervariable regions identified in the present study, only two intergenic regions (*ndh*J-*trn*F, *atp*H-*atp*F) have ever been used as genetic markers in previous studies, i.e., phylogenetic analyses of *Indigofera* (Zhao and Gao, 2015), and population genetic analyses of both the *I. bungeana* (Zhao et al., 2017) and *I. decora* complexes (Peng et al., 2015). Although most genes in the cp genomes of *Indigofera* were observed to be conserved in the present study, the intergenic spacers of *trn*K-*rbc*L, *ndh*J-*trn*F, *trn*L-*rps*4, *ycf*3-*psa*A, *atp*I-*atp*H, *atp*H-*atp*F, *psb*K-*rps*16, *pet*A-*psb*J, *trn*W-*psa*J, *ndh*F-*trn*L, and *ndh*G-*ndh*I, and introns of *ycf*1, *rpo*C2 exhibited divergence and were identified as potential biomarkers to indicate species (**Table 3** and **Figure 3**). The intergenic regions of *trn*K-*rbc*L and *ndh*F-*trn*L, which were predicted to have the highest nucleotide diversity, could be used as candidate DNA barcodes for fast species identification in *Indigofera*. In addition, the *ycf*1 gene, a large open reading frame, is commonly detected in land plant plastomes and is generally considered to play crucial roles in plant development (Drescher et al., 2000; Martinez-Alberola et al., 2013). The high variation rate of the *ycf*1 gene is valuable for intrageneric phylogenetic reconstruction, and it is comparable to the *mat*K in terms of consistency (Neubig et al., 2009; Dong et al., 2015). Therefore, *ycf*1 has been used as a potentially promising genetic marker in *Astragalus* L. (Dastpak et al., 2018; Zaveska et al., 2019), Orchidaceae (Whitten et al., 2014), and *Pinus* L. (Olsson et al., 2018). Additionally, *ycf*1 has also proposed to be a plastid candidate barcode in Papilionoideae, such as in *Sophora* (Liao et al., 2021), *Dalbergia* (Li et al., 2022b), *Onobrychis* Mill. (Moghaddam et al., 2022) and *Quercus* L. (Yang et al., 2017), etc. Consistent with these observations, we found that *ycf*1 exhibited high nucleotide diversity and could be used as for potential chloroplast marker for phylogenetic and evolutionary studies of *Indigofera* species.

In addition to highly variable regions, SSRs that are widely distributed in the genome are regarded as powerful tools for many aspects of evolutionary population biology, given their polymorphism (Tautz and Renz, 1984; Li et al., 2002). SSRs developed from transcriptome or genome have been used in *Indigofera* (Guo et al., 2016; Otao et al., 2016; Chikmawati et al., 2019; Royani et al., 2022). Chloroplast SSRs are typically non-recombinant, uni-parentally inherited, and effectively haploid, and as such have been widely used to fully understand the population genetic diversity and evolutionary history with biparental inherited molecular markers (Echt et al., 1998; Provan et al., 2001; Hatmaker et al., 2018; López-Villalobos and Eckert, 2018; Salmona et al., 2020; Yao et al., 2021). The abundant cp SSRs identified in the present study laid the foundation for the identification of assays detecting polymorphisms at the population level of *Indigofera*.

### 4.3 Phylogenetic implications

The monophyly and phylogenetic placement of the genus *Indigofera* was evaluated. The plastid phylogenomic analyses generated a highly resolved phylogeny in the present study. Species of the Genistoid clade formed the most basal clade, followed by the Mirbelioid clade. The IRLC and Robinioid clade formed a monophyletic clade (**Figure 5**). Within the sampled species, *Indigofera* species showed a close relationship with the Millettioid clade, consistent with the previous studies (Cardoso et al., 2013; de Queiroz et al., 2015; Oyebanji et al., 2020; Zhang et al., 2020b; Choi et al., 2022). However, phylogenetic relationships of the Robinioid clade and Genistoid clade in the complete plastome-based and PCGs-based phylogenetic trees exhibited different topologies (**Figure 5** and **Supplementary Figures S2, S3**). In addition, both the ML and BI trees constructed based on complete plastomes suggested that species of the Genistoid and Robinioid clades clustered together, except for *Templetonia retusa* (Vent.) R. Br. and *Lotus japonicus* (Regel) K. Larsen, which was inconsistent with the previous results (Zhang et al., 2020b; Choi et al., 2022). Rapidly evolution sites that tend to accumulate non-phylogenetic signals and potentially misaligned loci in the plastome dataset are often suggested to explain discordance (Burleigh and Mathews, 2004; Rodriguez-Ezpeleta et al., 2007; Philippe et al., 2011; Zhang et al., 2017). Copy and loss of gene duplication and lineage sorting can cause inconsistencies between gene trees, which in turn leads to conflicts between gene trees and species trees (Swenson and El-Mabrouk, 2012; Goncalves et al., 2019; Walker et al., 2019; Wei et al., 2021). It has been reported that a fewer misplaced leaves in the gene tree can lead to a completely different history, with significantly more duplications and losses (Hahn, 2007; Fang et al., 2010; Doroftei and El-Mabrouk, 2011).

The monophyly of *Indigofera* and its four clades was all strongly supported based on datasets of the complete cp genome and PCGs. However, the phylogenetic relationships of the Tethyan, East Asian, and Palaeotropical clades were inconsistent with nuclear ITS-based phylogenies (Schrire et al., 2003, 2009; Zhao, 2016). Within the Pantropical clade, sampled species exhibited a pantropical, Sino-Japanese, or Sino-Himalayan distribution consistent with previous studies (Schrire et al., 2009; Zhao, 2016). Species in this clade formed five monophyletic lineages, where *I. hirsuta*, a species with a widespread pantropical distribution, was the earliest diverging taxon. Three species (*I. carlesii*, *I. decora*, and *I. kirilowii*) of the *I. decora* complex, which were previously assigned to subsect. *Decorae*, formed a robustly supported clede, which is consistent with previous classifications (Fang and Zheng, 1994; Peng et al., 2015). These three species have shared numerical characteristics, with leaves and flowers significantly larger than other species of *Indigofera*, e.g. leaflets 1.5—6.5 (—7.5) × 1—3.5 cm, flowers 1.2—1.5 (—1.8) × 0.7—0.9 cm, wings and keels ca. 1.2—1.4 cm, and specifically distributed in eastern, central, and northern China, and extending to Korea and Japan (Fang and Zheng, 1994; Peng et al., 2015). Interestingly, four species (i.e., *I. atropurpurea*, *I. cassioides*, *I. stachyodes*, and *I. hebepetala* var. *glabra*) grouped closely in our phylogeny, which is inconsistent with the morphology-based classification (Fang and Zheng, 1994). Fang and Zheng (1994) classified *I. atropurpurea* and *I. hebepetala* var. *glabra* into subsect. *Bracteatae* based on the erect recemes and conspicuous bracts at anthesis, whereas *I. cassioides* and *I. stachyodes* were classified into subsect. *Pendulae* because their racemes are both pendulous and much longer than leaves. In addition, three Sino-Himalayan endemics (i.e., *I. franchetii*, *I. pendula*, and *I. scabrida*) showed close relationships. As a global biodiversity hotspot, the temperate Sino-Himalayan region harbors high levels of species richness (ca. 105 species) and morphological diversity of *Indigofera* (Fang and Zheng, 1994; Schrire et al., 2009; Gao and Schrire, 2010), and comprehensive phylogenetic relationships of *Indigofera* species in this region need a comprehensive sampling in the further study. Within the Palaeotropical clade, our samples involved only one species (*I. wightii*) distributed in tropical Asia. Within the East Asian clade, two species of subsect. *Pseudotinctoriae* (i.e., *I. bungeana* and *I. amblyantha*) showed close affinities, as reported in previous studies (Zhao, 2016; Zhao et al., 2017). Within the Tethyan clade, *I. linifolia*, *I. linnaei*, and *I. miniata*, which are mainly distributed in the tropics of Asia, Africa, Australia, and America, are all herbs with simple or 1-foliate leaves, or compound leaves with alternate leaflets, and keel beard loss, and their close affinity was also supported the previous phylogenetic studies based on ITS sequences (Schrire et al., 2009; Zhao, 2016). Overall, most of the *Indigofera* lineages were resolved with high support values in this study, which indicated that the chloroplast genome may be a suitable component in the construction of a robust phylogeny for *Indigofera*, and therefore represent an effective tool for resolving taxonomic controversy in this genus.

### 4.4 The adaptive evaluation analysis of *Indigofera* plastomes

Positive selection is thought to play a key part in the adaptation of organisms to diverse environments (Moseley et al., 2018; Feng et al., 2022b), however, purifying selection is more common than positive selection due to the constant elimination of deleterious mutations (Ogawa et al., 1999; Wu et al., 2020; Huang et al., 2021). The low Ka/Ks ratios within *Indigofera* species suggested that most genes are undergoing purifying selection to retain conserved functions in *Indigofera* species.

This analysis of adaptive evolution contributes to a deep understanding of genetic variation and changes in protein structure and function (Wicke et al., 2014; Li et al., 2022d). To investigate the differences in selective pressures between two different evolutionary branches of *Indigofera* and non-*Indigofera*, the PAML v4.10.6 package (Yang, 1997, 2007) was used to analyze selective pressures in a branch-site model for 70 shared PCGs. Thirteen genes with significant posterior probabilities for codon sites were identified in the BEB test. Codon sites with higher posterior probabilities can be considered as sites undergoing positive selection (Yang et al., 2005; Tyagi et al., 2020). These genes included two photosystem subunit genes (*psa*A and *psa*B), four NADH-dehydrogenase subunit genes (*ndh*A, *ndh*E, *ndh*F, and *ndh*I), two subunits of cytochrome b/f complex genes (*pet*A and *pet*N), two DNA-dependent RNA polymerase genes (*rpo*B and *rpo*C1), and the *acc*D, *ccs*A, and *mat*K genes.

Four genes (*ndh*A, *ndh*E, *ndh*F, and *ndh*I) encode a subunit of the NADH dehydrogenase complex and were responsible for the electron transport chain necessary to generate ATP during photosynthesis (Weiss et al., 1991; Kofer et al., 1998; Green, 2011; Peng et al., 2011; Zhang et al., 2020c; Xie et al., 2021). Previous studies have suggested that the *ndh* gene family may be involved in the protection of chloroplasts against photooxidative stress (Martin et al., 1996) and that the antioxidant stress capacity of plants is also closely related to their resistance to other environmental conditions (Shaaltiel et al., 1988). Species of *Indigofera*, which are mainly distributed in tropical and subtropical regions (Schrire et al., 2009; Peng et al., 2015; Zhao and Gao, 2015; Zhao, 2016), generally have strong heat tolerance and adaptability, and adapt to dry and hot environments by producing smaller leaves and fewer leaflets (Schrire et al., 2009; Niu et al., 2020; Zhao et al., 2020). In drought habitats, plants will reduce water transpiration by reducing leaf area, and this reduction of leaf area has a necessary trade-off of photosynthetic capacity (Peng et al., 2007; Niu et al., 2020). Environmental differences had a greater effect on the leaf blade phenotype of some species of *I. bungeana* complex (Niu et al., 2020; Zhao et al., 2020). We speculate that chloroplast functional genes involved in plant photosynthesis may play a key role in the ecological adaptation of *Indigofera* species to drought stress. In addition, *pet*A and *pet*N are membrane components necessary for the transport of respiratory and photosynthetic electrons (Gray, 1992; Yamori and Shikanai, 2016). Notably, the posterior probability value for the amino acid sites of *pet*N was as high as 0.968 in this study, indicating that *pet*N was strongly positively selected. Moreover, *acc*D is known to code the β-carboxylase subunit of acetyl-CoA carboxylase, which is essential for leaf growth and development (Madoka et al., 2002; Kode et al., 2005; Rousseau-Gueutin et al., 2013; Tyagi et al., 2020; Chen et al., 2021; Li et al., 2022a). Positive selection pressure on *accD* might be the adaptive evolution of *Indigofera* species to selection pressures imposed by their herbivores and pathogens (Schrire, 2013). We also identified positively selected sites in the *rpo*B, *rpo*C1, and *mat*K, which might have played key roles in the adaptive evolution of *Indigofera* species (Hao et al., 2010; Hertel et al., 2013; Shi et al., 2019). In summary, 13 PCGs showed significant positive selection markers, but the adaptive evolution of *Indigofera* in specific ecological environments needs to be further explored through molecular, physiological, and ecological studies.

## 5 Conclusion

We provided insights into the structural variation of the chloroplast genomes as well as the phylogenetic relationships in the genus *Indigofera* in the present study. To better perform phylogenetic construction, population genetics, and species identification for *Indigofera*, we screened promising molecular markers both from intergenic and coding regions. The monophyly of *Indigofera* and its four monophyletic clades was supported using a robust plastid phylogenomic framework. We found that cp genome data was effective in improving the resolution of phylogenies in *Indigofera*. Conflicting topologies were detected by complete cp genome and PCGs sequences. These topological incongruences deserve further exploration of the underlying biologically relevant evolutionary history, using nuclear and plastome datasets. We will also expand the genomic sampling to analyze the phylogenetic relationships, and biogeography of *Indigofera* in future studies.

## Data availability statement

The datasets presented in this study can be found in online repositories. The names of the repository/repositories and accession number(s) can be found below: https://www.ncbi.nlm.nih.gov/genbank/, OQ134123, OQ147467, OQ147468, OQ147469, OQ147470, OQ147481, OQ147471, OQ147466, OQ147482, OQ147472, OQ147473, OQ147474, OQ147475, OQ147476, OQ147477, OQ147478, OQ147479, OQ147480.

## Author contributions

S-MZ and X-LZ conceived and designed the study. S-MZ, FW, X-LZ and X-FG collected the samples. S-MZ, FW and S-YY performed the experiments. S-MZ analyzed the sequence data and drafted the manuscript. X-FG, Z-MZ and X-LZ participated in data analysis and revised the manuscript. All the authors contributed to and approved the submitted version.
