# Supplementary material for: Phylogenomics and plastome evolution of Indigofera (Fabaceae)
Source: Front Plant Sci. 2023 Jun 6;14:1186598. doi: 10.3389/fpls.2023.1186598 (PMC10280451; doi:10.3389/fpls.2023.1186598)
Supplement: Supplementary file 2 [file DataSheet_1.docx]

Supplementary Material

# Supplementary Figures and Tables

## Supplementary Tables

(See Data Sheet 1)

Table S1 List of 17 outgroups used in phylogenomic analyses.

Table S2 Raw output information of DnaSP for aligned sequence.

Table S3 Summary statistics of types of SSRs identified in each species.

Table S4 Summary statistics of the distribution of SSRs identified in each species.

Table S5 SSR comparison of 20 plastome genomes in *Indigofera*.

Table S6 Summary statistics of long repeat sequences in each species.

Table S7 Distribution of long repeat sequences of each species.

Table S8 Number of long repeat sequence of different lengths in each species.

Table S9 Summary of Pairwise Ka/Ks ratios in *Indigofera* and allied species.

## Supplementary Figures


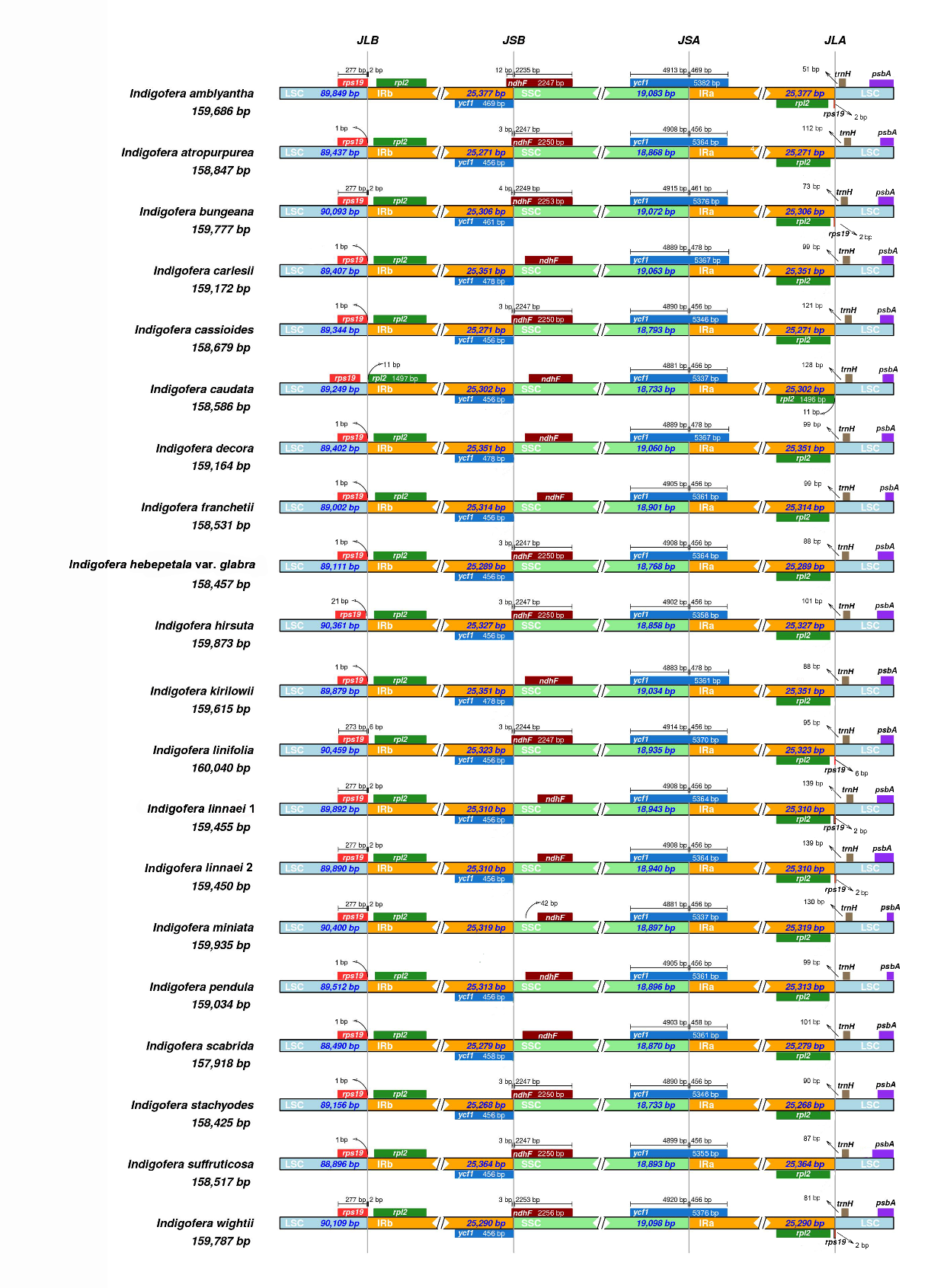


**Supplementary Figure S1.** Comparison of contraction and expansion of IR regions in the 20 cp genomes of *Indigofera*.


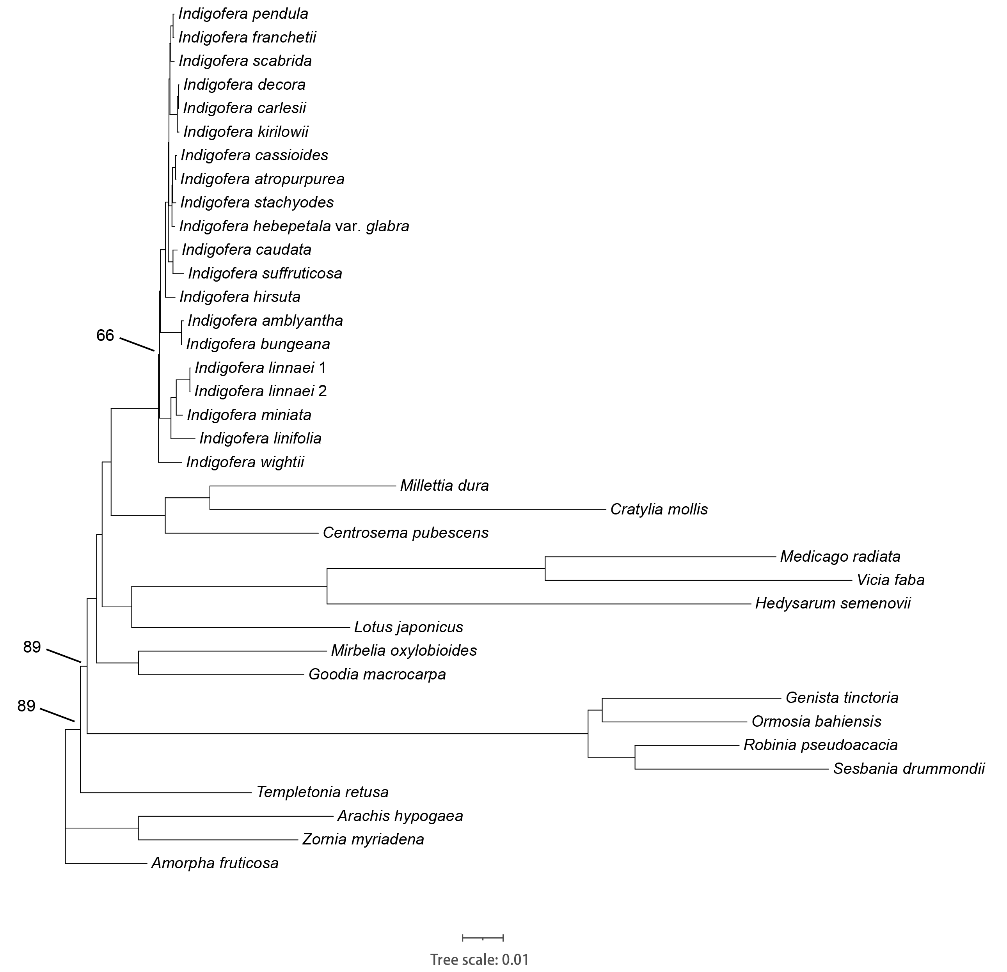


**Supplementary Figure** **S2.** Phylogenetic tree obtained using the maximum likelihood (ML) method of the complete cp genomes. The full support values are not indicated.


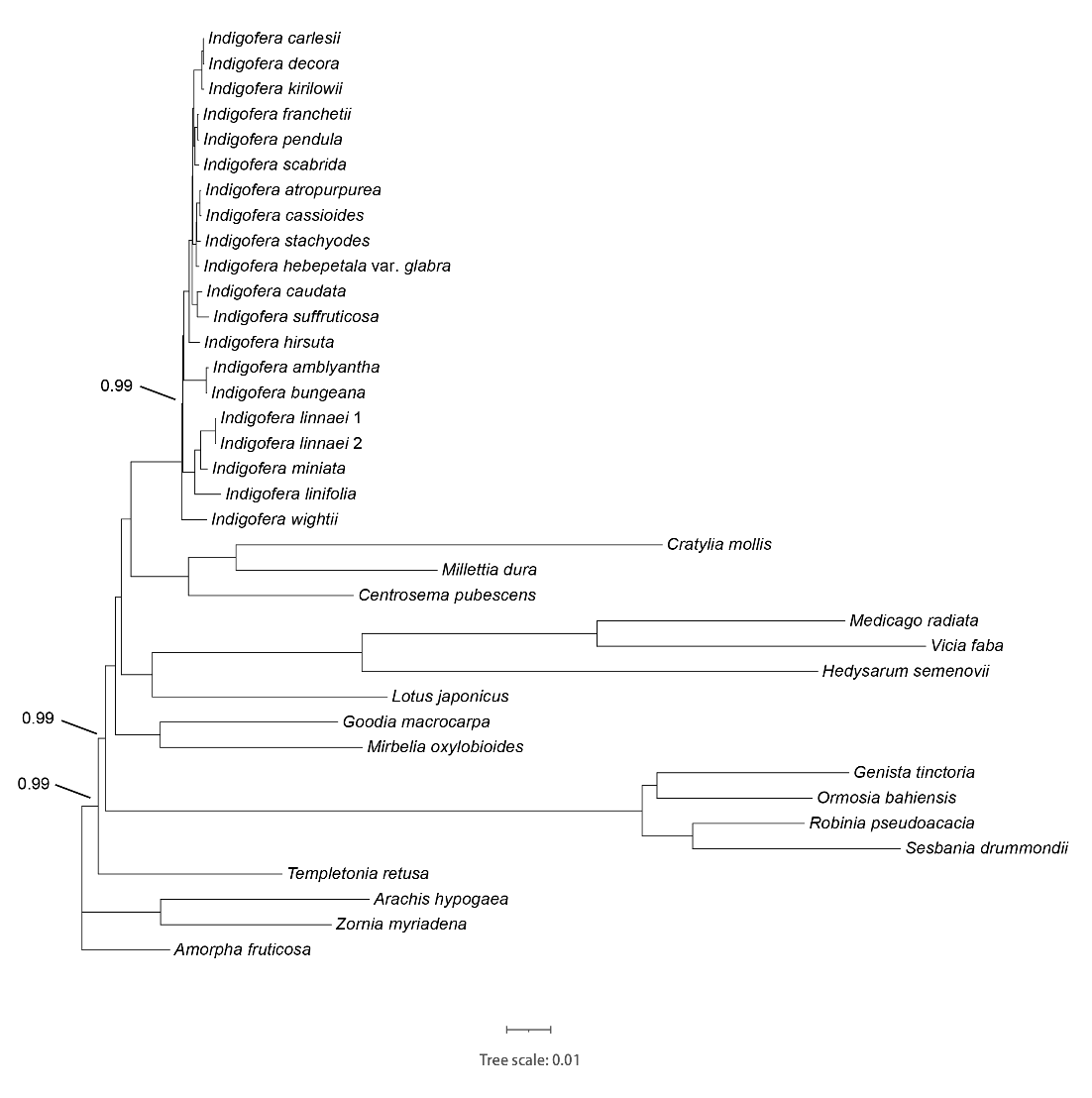


## Supplementary Figure S3. Phylogenetic tree obtained using the Bayesian inference (BI) method of the complete cp genomes. The full support values are not indicated.
